# Novel mouse strains to study circulating permeability factor(s) in primary focal segmental glomerulosclerosis

**DOI:** 10.1371/journal.pone.0274959

**Published:** 2022-09-22

**Authors:** Dirk den Braanker, Rutger Maas, Naomi Parr, Jeroen Deegens, Bart Smeets, Jack Wetzels, Johan van der Vlag, Tom Nijenhuis

**Affiliations:** 1 Department of Nephrology, Radboud Institute for Health Sciences, Radboud University Medical Center, Nijmegen, The Netherlands; 2 Department of Nephrology, Radboud Institute for Molecular Life Sciences, Radboud University Medical Center, Nijmegen, The Netherlands; 3 Department of Pathology, Radboud Institute for Molecular Life Sciences, Radboud University Medical Center, Nijmegen, The Netherlands; University of Utah School of Medicine, UNITED STATES

## Abstract

Recurrence of proteinuria after kidney transplantation in primary focal segmental glomerulosclerosis (FSGS) is unpredictable. Several putative circulating permeability factors (CPFs) have been suggested, but none have been validated. A clinically relevant experimental model is required that demonstrates the presence of CPF(s) in patient material, to study CPF(s) and possibly predict recurrence in patients. We aimed to develop a FSGS-prone Thy-1.1 transgenic mouse model with accelerated proteinuria after injection of samples from patients with FSGS. The Thy-1.1 transgene was backcrossed into 5 mouse strains. The age of onset and severity of spontaneous proteinuria varied between the different genetic backgrounds. 129X1/Sv^Thy-1.1^ and 129S2/SvPas^Thy-1.1^ mice displayed proteinuria at 4 weeks, whereas Balb/c^Thy-1.1^ and C57BL/6J^Thy-1.1^ mice developed proteinuria from 6 weeks, and were used further. We determined the maximum protein dose that could be injected without causing protein overload in each background. Balb/c^Thy-1.1^ and C57BL/6J^Thy-1.1^ males and females were injected with presumably CPF-containing plasmapheresis effluent from 6 FSGS patients, which induced albuminuria particularly in Balb/c^Thy-1.1^ males. Unfortunately, no response could be detected when using sera instead of plasmapheresis effluent, serum being more clinically relevant in the context of predicting FSGS recurrence. Considering the differences between responses elicited by serum and plasmapheresis effluent, simultaneously collected serum, plasma, and plasmapheresis effluent were tested. Whereas we could detect responses using a validated *in vitro* model, none of these presumably CPF-containing samples induced proteinuria in Balb/c^Thy-1.1^ males. Thus, we have extensively tested the Thy-1.1 mouse model on different genetic backgrounds with proteinuria after injection of FSGS patient material as clinically relevant readout. The Balb/c^Thy-1.1^ male mouse strain demonstrated the most promising results, but to detect CPF activity in FSGS serum e.g. prior to kidney transplantation, this strain clearly lacks sensitivity and is therefore not yet clinically applicable. It could, however, still be used as research tool to study CPFs in patient samples that did induce proteinuria.

## Introduction

Severe proteinuria is a hallmark of primary nephrotic syndrome (pNS). One of the main causes of pNS is primary focal segmental glomerulosclerosis (pFSGS). Immunosuppressive treatment can be successful, but patients are often steroid-resistant. Treatment by plasmapheresis (PP), thought to remove a presumed causative circulating permeability factor (CPF), can reduce proteinuria [1, 2]. But ultimately, patients often require dialysis or kidney transplantation. Proteinuria recurs in many of the transplanted kidneys within 24 hours, and indicates recurrence of the original disease (recurrent FSGS) [3, 4]. Prediction of recurrence is currently not possible, especially because the CPF(s) responsible for glomerular injury in pNS have not yet been identified, and since there is no method that detects the presence of a CPF in patient plasma prior to transplantation (reviewed in [5–8]).

The glomerular filtration barrier is composed of podocytes at the urinary side, glomerular endothelial cells at the blood side, and the glomerular basement membrane between these two cell types. In addition, mesangial cells are present that directly interact with glomerular endothelial cells. The glomerulus is encapsulated by Bowman’s capsule that is lined with parietal epithelial cells and connected to the tubules. CPF(s) may disturb important interactions between endothelium, epithelium, and mesangium, thereby causing proteinuria. We recently reported an *in vitro* model that is able to detect responses to presumably CPF-containing PP effluents [9]. However, *in vitro* models do not fully replicate the glomerular complexity. Therefore, an animal model with high sensitivity to glomerular injury and a clinically relevant readout, e.g. proteinuria, would be very instrumental.

The Thy-1.1 transgenic mouse carries a mouse-human chimeric transgene and expresses the Thy-1.1 protein on the surface of podocytes. The Thy-1.1 transgenic mouse has been used to study proteinuria and FSGS, both as a spontaneous model and an accelerated model, after administration of a monoclonal antibody against the Thy-1.1 protein [10–15]. Injection of anti-Thy-1.1 antibody induces acute albuminuria within 10 minutes, and FSGS lesions in most glomeruli within 3 weeks [12]. In the spontaneous model, proteinuria gradually develops at the age of 6–8 weeks [10]. Thus, glomeruli of Thy-1.1 mice appear to be more susceptible to the development of proteinuria and FSGS. Therefore, we hypothesized that Thy-1.1 mice could be suitable to detect the presence of CPF(s). Many studies have shown that the genetic background and gender of animals is crucial for their performance as model for various kidney diseases, including FSGS [16–29]. Accordingly, a Thy-1.1 model with a genetic background that demonstrates high sensitivity to CPF-containing plasma, with minimal variation in its clinically relevant outcome measure, could be a crucial asset in research and clinical practice.

In this study, we aimed to establish a novel Thy-1.1 mouse model, to be eventually applied to detect the presence of CPF(s) in biological material from patients with pFSGS. We evaluated the development of spontaneous proteinuria in Thy-1.1 mice with different genetic backgrounds and gender. Next, we analyzed the response of selected Thy-1.1 strains to administration of initial PP effluent from PP-responsive patients with (recurrent) FSGS, presumed to contain CPF(s). We used proteinuria as a clinically relevant and non-invasive readout. The most sensitive and robust Thy-1.1 strain was applied to assess whether, in addition to PP effluent, serum from patients with pNS with presumed presence of CPF(s) could also induce proteinuria.

## Materials and methods

### Patient and control material

Use of patient material was approved by the regional medical-ethical committee Arnhem-Nijmegen, under file number 09/073, and has been carried out in compliance with the Helsinki declaration. All human participants signed informed consent prior to the study. Clinical data of the patients whose materials were used in this study are summarized in Table 1. PP effluent was obtained from initial PP session of five patients with recurrence of FSGS after transplantation (rec1-5) and from one PP-dependent patient with FSGS in the native kidneys (nat1). A pool of citrated plasma from five healthy donors was used as control plasma. Recurrent FSGS sera were collected during active disease from post-transplantation recurrence patients, prior to the first PP treatment after recurrence (rec1B, rec7, rec8), and/or before transplantation (rec1A, rec2, rec5, rec7A). Serum from one patient with recurrent FSGS (rec6) who did not clinically respond to PP therapy was collected before the eighth PP treatment after transplantation. Sera were taken from two patients with FSGS in the native kidney who were not yet transplanted, but were PP-responsive (nat2, nat3). Control sera were obtained from three healthy donors and from patients in whom proteinuria did not recur after transplantation, either after (non-rec1-4) or before (non-rec5) transplantation. Additionally, simultaneously collected pre-PP serum, (heparin) plasma, and PP effluent was used from two recurrent FSGS patients post-transplantation (rec6 and rec7), and from one PP-responsive FSGS patient with FSGS in the native kidneys (nat3). Serum and plasma from a patient with FSGS in the native kidneys (not treated with PP) was collected during active disease and during (partial) remission (nat4).

**Table 1:** Clinical characteristics of patients.

Patient code	Sample code and material		Gender	Age at diagnosis	Age at sampling time	Transplant number	Timing of sample	Onset of recurrence after TX	Serum creatinine (μmol/L)	Serum albumine (g/L)	Proteinuria (g/10mmol creat)	Immunosuppression at sampling time
Healthy	Healthy	pooled plasma	3F,2NA	NA	NA	NA	NA	NA	NA	NA	NA	None
healthy1	healthy1	serum	M	NA	29	NA	NA	NA	NA	NA	NA	None
healthy2	healthy2	serum	M	NA	25	NA	NA	NA	NA	NA	NA	None
healthy3	healthy3	serum	F	NA	27	NA	NA	NA	NA	NA	NA	None
rec1	rec1A	serum	M	20	20	pre-TX	ESRD	Day 3	76	13	13.4 g/day	Pred
	rec1B	serum			24	1	prior to PP1 post-TX		200	18	5.4	Tacro, MMF, Pred
	rec1	PP effluent					PP1 post-TX					
rec2	rec2	serum	M	53	68	pre-TX	Relapse	Day 3	204	26	10	None (pre-TX sample)
	rec2	PP effluent			73	pre-TX	PP1 pre-TX (1.5y later)		742	22	7.5	None (pre-TX sample)
rec3	rec3	PP effluent	F	26	39	1	PP1 post-TX	Day 1	146	27	20.0	Tacro, MMF, Pred
rec4	rec4	PP effluent	F	10	32	3	PP1 post-TX	Day 1	678	40	9.0	Rtx pre-TX, Tacro, MMF, Pred
rec5	rec5	serum	M	7	20	pre-TX	1 day pre-TX	Day 2	1038	44	Anuric	None (pre-TX sample)
	rec5	PP effluent			20	1	PP1 post-TX		127	24	23.0	Tacro, MMF, Pred
rec6	rec6	serum	M	23	26	1	prior to PP8 post-TX	Day 2	673	34	7.1	Tacro, MMF, Pred
rec7	rec7A	serum	M	60	61	pre-TX	1 month before PP1	Day 2	193	20	11.6	Pred
	rec7	serum			65	1	prior to PP1 post-TX		467	30	4.2	Tacro, MMF, Pred
	rec7	plasma					prior to PP1 post-TX					
	rec7	PP effluent					PP1 post-TX					
rec8	rec8	serum	M	38	49	1	prior to PP1 post-TX	Day 1	175	28	4.0	Bas, Tacro, MMF, Pred
	rec8	plasma					prior to PP1 post-TX					
	rec8	PP effluent					PP1 post-TX					
nat1	nat1	PP effluent	M	15	18	No TX	PP1	No TX	459	9	7.3	Mpred
nat2	nat2	serum	M	55	58	No TX	ESRD (dial)	No TX	NA	NA	Anuric	None
nat3	nat3	serum	M	69	69	No TX	prior to PP1 (AKI/dial)	No TX	674	19	3.4	Pred
	nat3	plasma					prior to PP1 (AKI/dial)					
	nat3	PP effluent					PP1					
nat4	nat4 (act)	plasma	F	59	59	No TX	At diagnosis	No TX	169	18	8.6	None
	nat4 (act)	serum										
	nat4 (rem)	plasma			60		SRNS (partial Pred responsiveness)		112	28	4.8	MMF, Pred
	nat4 (rem)	serum										
non-rec1	non-rec1	serum	M	43	68	1	2 years post-TX	No rec	86	39	0.1	Tacro, Pred
non-rec2	non-rec2	serum	M	63	74	1	2 years post-TX	No rec	152	NA	0.4	Tacro, MMF, Pred
non-rec3	non-rec3	serum	M	18	27	1	2 months post-TX	No rec	177	41	0.1	Tacro, MMF, Pred
non-rec4	non-rec4	serum	M	18	27	1	1 day post-TX	No rec	224	34	0.4	Tacro, MMF, Pred
non-rec5	non-rec5	serum	F	8	23	1	1.5 years pre-TX	No rec	311	38	0.7	None (pre-TX sample)

Act: active FSGS; AKI: acute kidney injury; Bas: Basiliximab; dial: dialysis; ESRD: end-stage renal disease; MMF: Mycophenolate; Mpred: Methylprednisone; nat: FSGS in the native kidneys; non-rec: non-recurrent FSGS (FSGS patient without post-transplant recurrence); PP: plasmapheresis; Pred: Prednisone; rec: recurrent FSGS; rem: remission; Rtx: Rituximab; SRNS: steroid-resistant nephrotic syndrome; Tacro: Tacrolimus; TX: transplantation; NA: not available. ‘A’ or ‘B’ is assigned to the sample code to indicate different samples from the same patient of the same type (serum), when used in the same figure.

Patients with FSGS in their native kidneys tested negative for mutations linked to glomerular diseases known at the time of sample collection. Besides serum, citrated or heparinized plasma and PP effluent were used in this study. Samples were aliquoted and frozen at -80°C immediately after collection. Aliquots were thawed and centrifuged at 5,000g for 5 min at 4°C to remove platelets and cellular debris. Protein concentration was determined using BCA protein assay (Sigma). For animal experiments, samples were concentrated using a SpeedVac Concentrator (Savant SC210A) or diluted in phosphate-buffered saline (PBS) to achieve appropriate protein concentrations.

### Animals

Thy-1.1 transgenic C57BL/6J mice [12] were backcrossed five times with wild-type FVB/NCrl, 129S2/SvPasCrl (both from Charles River Laboratories, Sulzfeld, Germany), Balb/cJRj (Janvier, Le Genest-Saint-Isle, France), or 129X1/SvJ (Jackson, Bar Harbor, USA) mice. After crossing generation F5, breeding couples consisted of a heterozygous Thy-1.1 transgenic mouse and a wild-type from the same strain. The presence of the transgene was determined by polymerase chain reaction (PCR) on genomic DNA from ear punches, with forward primer 5’-CGCCTGAGTCCTGATCTC-3’ and reverse primer 5’-AACCTGCATCTTCACTGG-3’, resulting in a specific 834 bp amplicon.

### Animal experiments

All animal procedures were approved by the Animal Ethical Committee of the Radboud University Nijmegen and of the Netherlands (CCD, AVD1030020174226). Experiments were performed at our animal facilities in accordance with the European Directive 2010/63/EU. To evaluate development of spontaneous proteinuria in the different genetic backgrounds, urine of Thy-1.1 transgenic mice of 4–18 weeks old was collected in an Eppendorf tube weekly. For experiments regarding exposure to patient material, Thy-1.1 transgenic animals at age 5 weeks received an intravenous injection of 200 μl sample in the tail vein and were placed in metabolic cages for 24 hours for urine collection. At the end of the experiment, mice were sacrificed by cervical dislocation. Urine albumin was determined using a radial immunodiffusion assay (Mancini) and creatinine was measured using a Cobas 8000 modular analyzer (Roche).

### Cell culture

Conditionally immortalized human podocytes (hPod) were originally obtained from M. Saleem as described in [30], and cultured at 33° C with 5% CO_2_, and differentiated at 37° C for 9 days on collagen-coated surface in RPMI Dutch-modified medium (Invitrogen), supplemented with 10% fetal calf serum (FCS), 1% glutamate, 1% insulin-transferrin-selenium, and 1% penicillin/streptomycin (Thermo Fisher Scientific) [30].

### Flow cytometry

Experiments were performed as previously published [9]. Differentiated hPod cultured in 12-well plates were treated in duplicate with 10% (v/v) patient plasma or serum (replacing FCS) in the presence of anticoagulants PPACK dihydrochloride (10 μM; Santa Cruz) and heparin (100 μg/ml; Sigma) for 24 hours. After treatment, cells were harvested via trypsinization, resuspended in medium with FCS, and collected using CytExpert software on a CytoFLEX flow cytometer (Beckman Coulter). Kaluza v1.5a (Beckman Coulter) was used for data analysis, after gating on live cells based on forward versus side scatter plots. Geometric mean of side scatter of cells exposed to patient plasma or serum was normalized to pooled healthy donor plasma or the mean of sera of three healthy donors, respectively.

### Statistical analyses

For animal experiments, groups of mice with the same genetic background that received different material were compared using one-way ANOVA with Dunnett’s multiple comparisons test and considered significantly different when p≤0.05. For the *in vitro* assay the same one-way ANOVA test was used by comparing to pooled plasma of five healthy donors. For comparison of the different mouse strains, positive responses to patient material were defined by an albumin/creatinine ratio exceeding the mean plus 3 times the standard deviation of proteinuria of healthy plasma-injected mice of the same strain. Data analysis was performed using GraphPad Prism version 5.03.

## Results

### Genetic background influences development of spontaneous proteinuria in Thy-1.1 mice

We aimed to establish a Thy-1.1 mouse model with proteinuria upon injection of patient material as readout, in which CPF-induced proteinuria is ideally detectable prior to the moment that mice develop spontaneous proteinuria. Therefore, we first evaluated development of spontaneous proteinuria by weekly monitoring of urinary albumin in different genetic backgrounds with the Thy-1.1 transgene (Table 2). Urinary albumin levels in Balb/c^Thy-1.1^ and C57BL/6^Thy-1.1^ mice started to increase at the age of 6 weeks (S1A–S1D Fig). Thus, for these strains, the age of injection was determined at age 5 weeks, approximately one week before spontaneous proteinuria starts to develop. The 129X1/Sv^Thy-1.1^ and 129S2/SvPas^Thy-1.1^ mice already displayed albuminuria at 4–5 weeks (S1E–S1H Fig), which rapidly increased during the following weeks. However, taking into account that all mice are weaned at age 3–4 weeks and required obligatory acclimatization for one week, the age of injection of 129X1/Sv^Thy-1.1^ and 129S2/SvPas^Thy-1.1^ mice was pragmatically also set to age 5 weeks. FVB/N^Thy-1.1^ breeding was discontinued because generation F1 mice already displayed severe proteinuria at the age of 4–5 weeks, which progressed to nephrotic syndrome (edema) at 9 weeks (S1I and S1J Fig), leading to death or sacrifice by reaching humane endpoints. We therefore continued with Balb/c^Thy-1.1^, C57BL/6^Thy-1.1^, 129X1/Sv^Thy-1.1^, and 129S2/SvPas^Thy-1.1^ mice.

**Table 2:** Development of spontaneous proteinuria in Thy-1.1 mice with different backgrounds.

Thy-1.1 mouse strain	Generation backcrossed with C57BL/6	Gender	Age of onset albuminuria (weeks)	Urine albumin at week 5 (μg/ml)
**Balb/c^Thy-1.1^**	F5	M, F	6	34 ± 11
**C57BL/6^Thy-1.1^**	F6, F7	M, F	6	172 ± 109
**129X1/Sv^Thy-1.1^**	F5, F6	M, F	4	2796 ± 1460
**129S2/SvPas^Thy-1.1^**	F7	M, F	4	4089 (n = 1)
**FVB/N^Thy-1.1^**	F1	M, F	4	12495 ± 5465

### Genetic background of Thy-1.1 mice influences proteinuria caused by protein overload after healthy plasma injection

**Fig 1:**
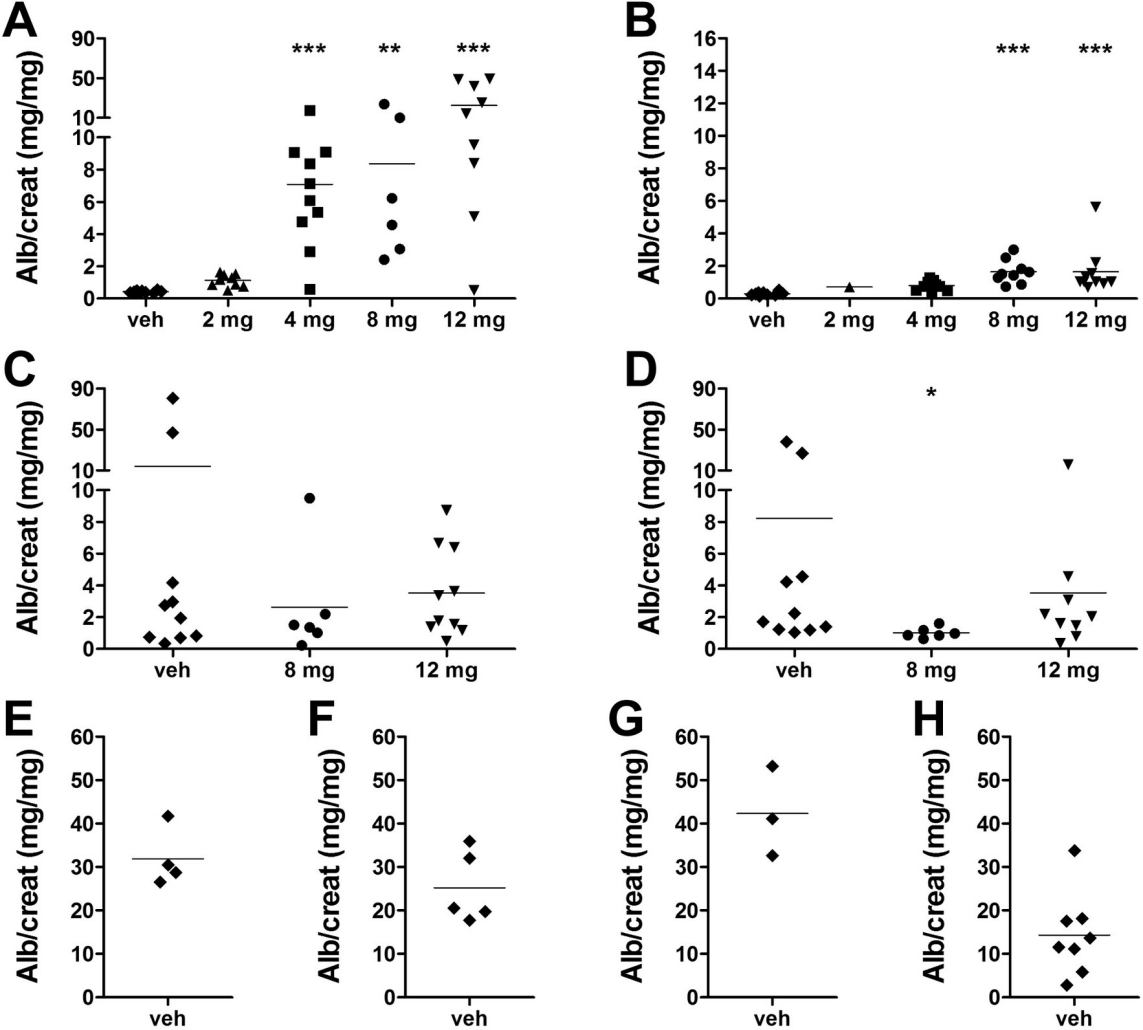
Different doses healthy plasma were injected in the Thy-1.1 mice with different backgrounds to determine maximum injection dose. (**A**) Balb/c^Thy-1.1^ males, (**B**) Balb/c^Thy-1.1^ females, (**C**) C57BL/6^Thy-1.1^ males, (**D**) C57BL/6^Thy-1.1^ females were injected intravenously with various doses healthy plasma protein at 5 weeks ± 3 days old. (**E**) 129X1/Sv^Thy-1.1^ males, (**F**) 129X1/Sv^Thy-1.1^ females, (**G**) 129S2/SvPas^Thy-1.1^ males, and (**H**) 129S2/SvPas^Thy-1.1^ females received PBS vehicle (veh) injection only. Individual animal data and group means of urinary albumin/creatinine (alb/creat, mg/mg) ratio are presented. One-way ANOVA: * p≤0.05, ** p≤0.01, *** p≤0.001 versus vehicle.

Injection of patient serum/plasma is known to induce proteinuria by itself, presumably due to protein overload. In order to prevent protein overload whilst being able to expose mice to the highest possible dose of CPF(s), appropriate plasma injection doses were determined. Balb/c^Thy-1.1^, C57BL/6^Thy-1.1^, 129X1/Sv^Thy-1.1^ and 129S2/SvPas^Thy-1.1^ mice received various doses of pooled plasma from 5 healthy donors, and albumin/creatinine ratio in 24 hour-urine was analyzed (Fig 1). A dose-dependent increase in proteinuria was observed in Balb/c^Thy-1.1^ males and females. Proteinuria of Balb/c^Thy-1.1^ males after injection of 2 mg healthy plasma protein was not significantly different from PBS vehicle injection (Fig 1A). Injection of 4, 8, and 12 mg healthy plasma protein induced proteinuria significantly higher than vehicle injection. Therefore, 2 mg was selected as injection dose for Balb/c^Thy-1.1^ males. Responses of female Balb/c^Thy-1.1^ mice to healthy plasma were weaker compared to males, and 12 mg was chosen as injection dose for Balb/c^Thy-1.1^ females (Fig 1B). In contrast to Balb/c^Thy-1.1^ mice, proteinuria after PBS vehicle injection already highly varied in C57BL/6^Thy-1.1^ males and females, pointing out variation in spontaneous proteinuria (Fig 1C and 1D). Since injection of 8 or 12 mg healthy plasma protein in C57BL/6^Thy-1.1^ males as well as females did not result in higher proteinuria than injection of vehicle, 12 mg was selected as plasma injection dose for C57BL/6^Thy-1.1^ mice. The 24 hour urinary albumin excretion was already high after vehicle injection in 129X1/Sv^Thy-1.1^ and 129S2/SvPas^Thy-1.1^ mice (Fig 1E–1H), in line with the finding of severe albuminuria at week 5 when weekly analyzing spot urines (Table 2 and S1E–S1H Fig). Therefore, 129X1/Sv^Thy-1.1^ and 129S2/SvPas^Thy-1.1^ mouse strains were excluded for further experimentation.

### Thy-1.1 Balb/c^Thy-1.1^ male mice display a strong response to (presumably CPF-containing) FSGS plasmapheresis effluents

**Fig 2:**
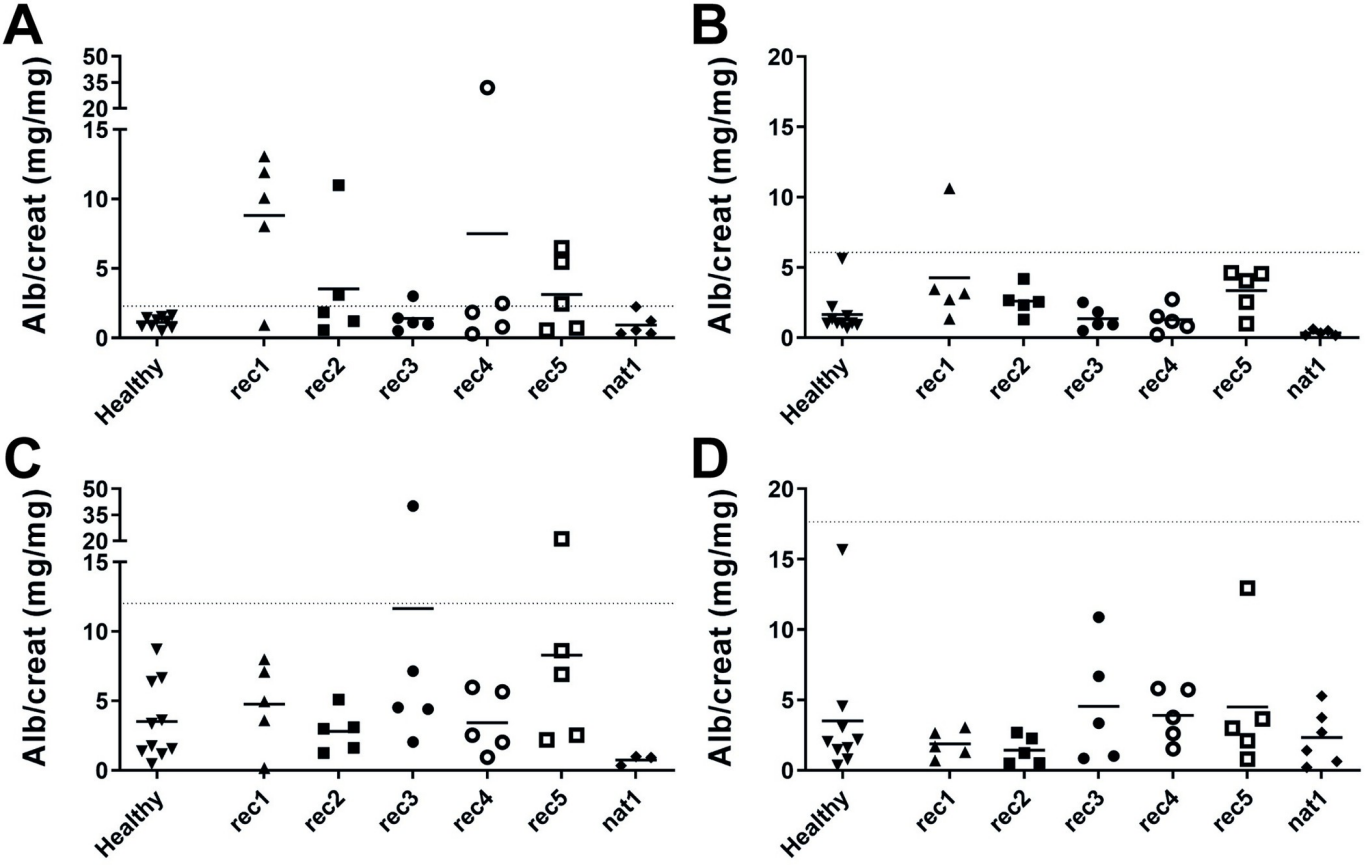
Proteinuria of Balb/c^Thy-1.1^ and C57BL/6^Thy-1.1^ mice induced by FSGS plasmas. (**A**) Balb/c^Thy-1.1^ males, (**B**) Balb/c^Thy-1.1^ females, (**C**) C57BL/6^Thy-1.1^ males, and (**D**) C57BL/6^Thy-1.1^ females were injected intravenously, at 5 weeks ± 3 days old, with 2 mg (Balb/c^Thy-1.1^ males), or 12 mg (Balb/c^Thy-1.1^ females, C57BL/6^Thy-1.1^ males and females) PP effluent protein from six patients with presumed CPF(s). Individual animal data and group means of urinary albumin/creatinine (alb/creat, mg/mg) ratio are presented. Data of healthy plasma injections from Fig 1 are shown for comparison with patient PP effluents. Mice were defined as responding to patient PP effluent if proteinuria exceeded the mean plus 3 times the standard deviation of proteinuria of healthy plasma-injected mice (indicated with dotted lines).

We used patient samples with a high likelihood of CPF(s) being present, such as PP effluent from FSGS patients whose PP treatment reduced proteinuria, to test in our selected mice strains (i.e. Balb/c^Thy-1.1^ and C57BL/6^Thy-1.1^ males and females). Six PP effluents presumed to contain CPF(s) (for clinical details of the patients, see Table 1) were injected and proteinuria response was analyzed (Fig 2). Table 3 displays the number of mice that responded to the patient PP effluents, as defined by an albumin/creatinine ratio exceeding the mean plus 3 times the standard deviation of proteinuria of healthy plasma-injected mice of the same strain. In Balb/c^Thy-1.1^ males (Fig 2A), PP effluent of rec1 induced proteinuria in 4 out of 5 mice. PP effluent from patient rec5 induced proteinuria in 3 out of 5 Balb/c^Thy-1.1^ males, PP effluent from patients rec2 and rec4 induced proteinuria in 2 out of 5 Balb/c^Thy-1.1^ males, and PP effluent from patient rec3 induced proteinuria in 1 Balb/c^Thy-1.1^ male. Balb/c^Thy-1.1^ females (Fig 2B), injected with a 6-fold higher dose, demonstrated minimal responses to the PP effluents. Variation, indicated by the standard deviation (SD), in proteinuria of healthy plasma-injected Balb/c^Thy-1.1^ females was slightly larger than in Balb/c^Thy-1.1^ males, and substantially less Balb/c^Thy-1.1^ females responded to PP effluents. The SD of proteinuria of C57BL/6^Thy-1.1^ males (Fig 2C) and females (Fig 2D) was high, and very few C57BL/6^Thy-1.1^ mice responded to PP effluents compared to Balb/c^Thy-1.1^ males. Overall, Balb/c^Thy-1.1^ male mice constituted the strain with the strongest responses and the lowest variation to presumably CPF-containing PP effluents.

**Table 3:** FSGS plasma induced proteinuria mostly in Balb/c^Thy-1.1^ males.

	healthy	rec1	rec2	rec3	rec4	rec5	nat1
**Balb/c^Thy-1.1^ males**	0/9	4/5	2/5	1/5	2/5	3/5	0/5
**Balb/c^Thy-1.1^ females**	0/10	1/5	0/5	0/5	0/5	0/5	0/5
**C57BL/6^Thy-1.1^ males**	0/10	0/5	0/5	1/5	0/5	1/5	0/3
**C57BL/6^Thy-1.1^ females**	0/9	0/5	0/5	0/5	0/5	0/5	0/6

The number of mice responding to PP effluent from FSGS patients, with proteinuria exceeding the mean plus 3 times the standard deviation of proteinuria of healthy plasma-injected mice, as shown in Fig 2.

### Sera from patients with primary FSGS do not induce proteinuria in Balb/c^Thy-1.1^ male mice

**Fig 3:**
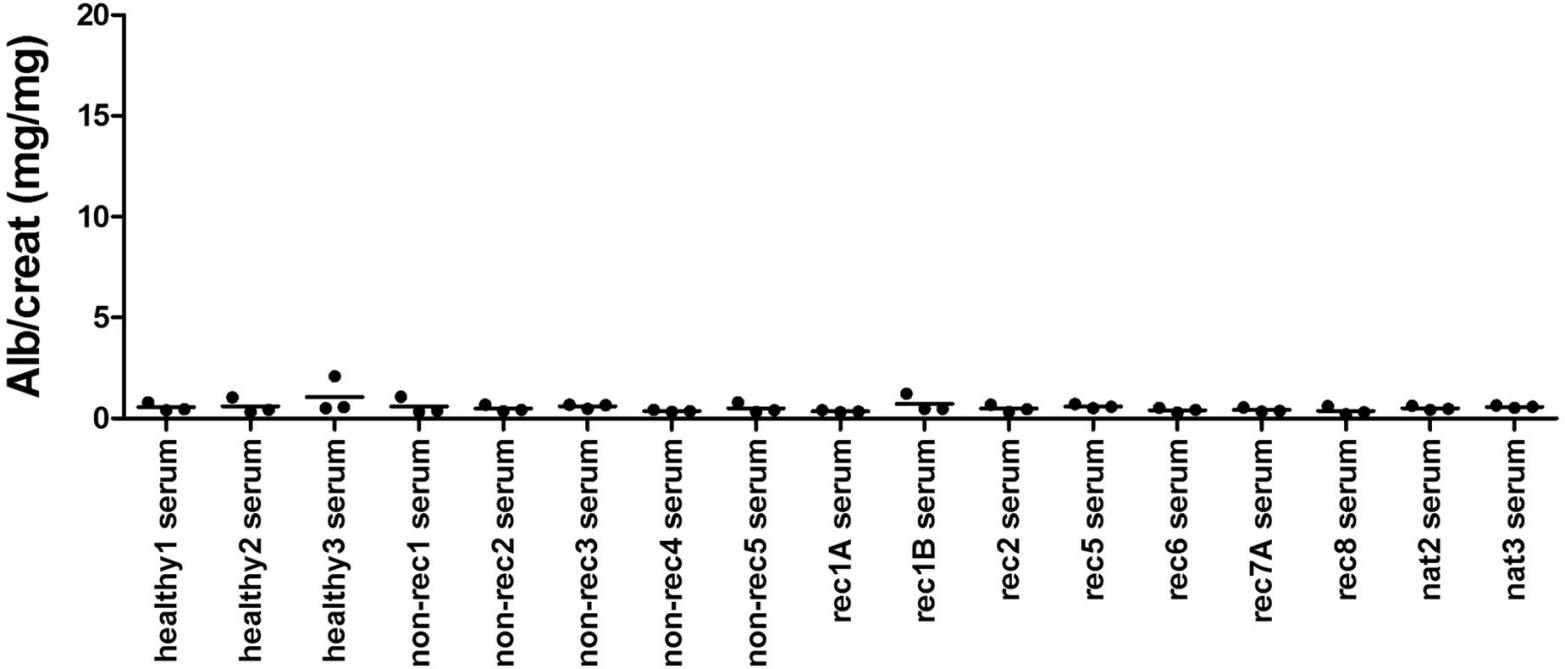
pNS sera did not induce proteinuria in Balb/c^Thy-1.1^ males. Balb/c^Thy-1.1^ males were injected intravenously, at 5 weeks ± 3 days old, with 2 mg serum protein from patients with recurrent FSGS, FSGS in the native kidneys, non-recurrent FSGS, or healthy controls. Individual animal data and group means of urinary albumin/creatinine (alb/creat, mg/mg) ratio are presented. No significant differences (p<0.05) compared to sera from healthy donors.

The Balb/c^Thy-1.1^ male mice showed the strongest responses with the lowest variation in tests with PP effluent plasma from FSGS patients during active (recurrent) disease, presumed to contain high levels of CPF(s), and as such could aid in future studies looking to identify culprit CPF(s). However, for clinical applicability as a model to predict the risk of recurrence prior to transplantation, serum or plasma taken from whole blood is the appropriate clinical material to screen. Therefore, sera from patients with recurrent FSGS, not yet transplanted PP-responsive patients with FSGS in the native kidneys, non-recurrent FSGS, and healthy controls were injected in Balb/c^Thy-1.1^ male mice (Fig 3). However, none of the recurrent FSGS or (active) native kidney FSGS sera induced proteinuria, whereas healthy control sera and sera from non-recurrent FSGS patients also had no effect.

### Simultaneously collected serum, plasma, and PP effluent samples do not induce a proteinuric response

**Fig 4:**
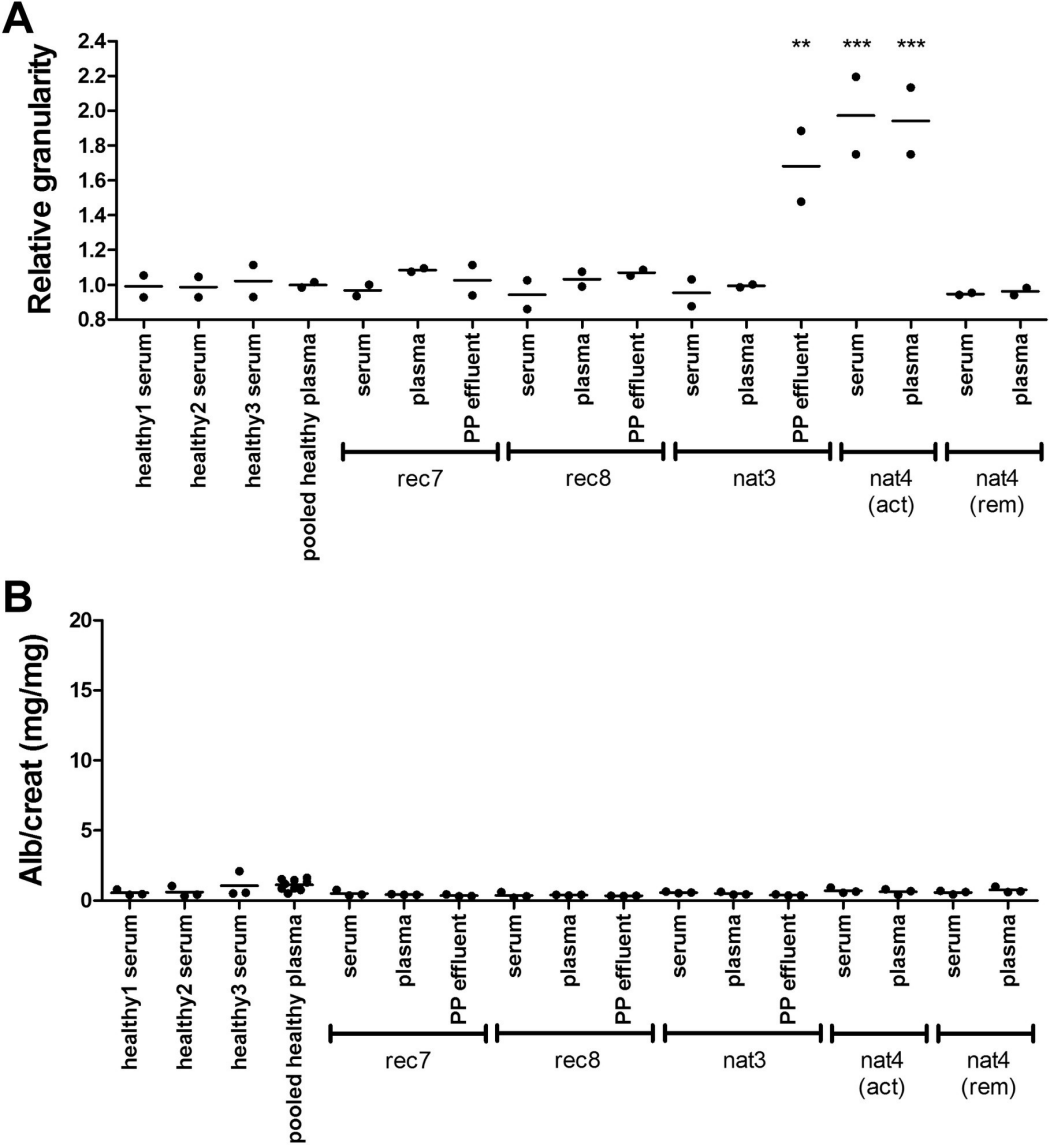
*In vitro* and *in vivo* responses to simultaneously collected serum, plasma, and PP effluent. (**A**) Cellular granularity of cultured human podocytes (hPod) exposed to simultaneously collected serum, plasma, and PP effluent, as measured by flow cytometry, is represented by the median side scatter relative to healthy controls. Relative granularity of two replicate experiments and their mean are presented. ** p<0.01, *** p<0.001 versus pooled plasma from five healthy donors for (PP) plasma and versus three healthy donor sera for sera. (**B**) Albuminuria of Balb/c^Thy-1.1^ males injected intravenously, at 5 weeks ± 3 days old, with 2 mg protein from serum, plasma, or PP effluent from three FSGS patients during active disease (rec7, rec8, nat3) and serum and plasma from one FSGS patient not treated with PP, during active disease and remission (nat4). Individual animal data and group means of urinary albumin/creatinine (alb/creat, mg/mg) ratio are presented. Data of healthy donor sera derived from Fig 3 and pooled healthy plasma derived from Fig 1 are shown for comparison with patient samples. Although none of the patient sera induced proteinuria, the ages of the patients in this study do not match the ages of the healthy donors, which may limit interpretation of these data.

The previous experiments suggested that Balb/c^Thy-1.1^ males responded to presumably CPF-containing PP effluent, but not to sera presumed to contain CPF. This result could theoretically be due to differences in CPF levels and/or activity between PP effluent and serum. To test this hypothesis, we simultaneously collected serum and plasma prior to PP and the PP effluent of the following PP treatment from three FSGS patients during active disease, and serum and plasma from a native FSGS patient not treated with PP, during active disease as well as during steroid-induced remission. In order to substantiate the presence of CPF(s) in these simultaneously collected samples, we first applied our previously published *in vitro* human podocyte assay using flow cytometry-quantified cellular granularity as readout, in which we have shown that some but not all presumably CPF-containing PP effluents induce a response [9]. Fig 4A shows that both serum and plasma from patient nat4 while showing active disease induced a response *in vitro*, whereas serum and plasma from the same patient at remission did not. Interestingly, PP effluent from patient nat3 induced granularity, but serum and plasma did not. Post-transplantation serum, plasma, and PP effluent from patients rec7 and rec8 did not induce granularity. Thus, these *in vitro* data showed that in three of the samples CPF activity could be detected with the use of our *in vitro* test model, and that discrepancies can indeed exist between serum/plasma and PP effluent. Next, the simultaneously collected serum, plasma, and PP effluent samples were injected in Balb/c^Thy-1.1^ males (Fig 4B). Despite *in vitro* responses to three of the samples, none of the samples induced proteinuria. Therefore, we cannot draw conclusions on potential differences in effect between serum, plasma, and PP effluent *in vivo*.

## Discussion

We aimed to establish a mouse model that would allow for detection of CPF(s) in the blood of patients with pFSGS. Our hypothesis was that FSGS-prone mice would develop accelerated proteinuria after injection of presumably CPF-containing patient samples. Such a mouse model could in the future aid in studying the identity of CPF(s), as well as screening pre-transplant sera from patients with pFSGS to predict recurrence. In our approach we first established and compared 5 different Thy-1.1 mouse strains. Then we tested PP effluents with presumed high concentration of CPF(s) in a selection of these strains. Our study demonstrated that genetic background and gender are key determinants of spontaneous proteinuria, protein overload proteinuria, and proteinuria induced by presumably CPF-containing PP effluents. In addition, determining the optimal dose of plasma protein for each strain specifically was essential. Compared to the other genetic backgrounds, Balb/c^Thy-1.1^ males were the most sensitive to presumably CPF-containing PP effluent and showed the lowest variation in proteinuria. Therefore, the Balb/c^Thy-1.1^ males may contribute to further studies identifying the CPFs in biological samples of patients that induce a detectable proteinuric response in this strain. However, for a model to have clinical value in actually predicting the presence of CPF(s) in patients with FSGS prior to transplantation, and thus the likelihood of post-transplantation recurrence, patient serum is the appropriate biological test sample. Unexpectedly, we could only induce proteinuria by injecting specific PP effluent, but not by serum or plasma. The failure of plasma or serum to induce proteinuria limits the clinical applicability of our Balb/c^Thy-1.1^ males at this moment.

Only a very limited number of studies have used animal models to detect CPF activity in PP effluent, serum or plasma from FSGS patients. Proteinuria of rats and rabbits injected with patient samples were used in the past and, more recently, zebrafish expressing a fluorescently-labeled plasma protein as an indicator for proteinuria [31–35]. Savin *et al*. developed an *ex vivo* model using freshly isolated glomeruli from rats [36]. A higher glomerular albumin permeability was measured after exposure to serum from patients with recurrent FSGS compared to control serum [37]. So far, none of the previously published animal or animal-derived models have been further developed for clinical application, for example to detect CPF in serum prior to transplantation in order to predict the risk of recurrence. We recently reported an *in vitro* assay using cultured human podocytes that was able to detect the presence of CPF(s) in PP effluent from patients with pFSGS [9]. However, we want to emphasize the importance of *in vivo* models, which more closely mimic the physiological and clinical situation and could be of added value to the currently available *in vitro* assays for studying and demonstrating the presence of CPFs in patient samples.

Many studies have shown the influence of genetic background on the performance of mouse models for nephrotic syndrome and other kidney diseases. C57BL/6 mice are relatively less susceptible to development of proteinuria, glomerulosclerosis, and hypertension [16, 20, 23, 29]. Injection of adriamycin can induce FSGS and proteinuria in Balb/c mice, but C57BL/6 mice are quite resistant [21]. Injection of similar doses of puromycin aminonucleoside induces higher proteinuria in Balb/c mice than in C57BL/6 mice [22]. Another study showed more pronounced glomerulosclerosis and albuminuria in 129Sv mice than in C57BL/6 mice after 5/6 nephrectomy [24]. Furthermore, induction of type I diabetes resulted in histological glomerular damage, proteinuria, increased oxidative stress, and hyperfiltration in 129Sv and Balb/c mice, whereas C57BL/6 mice only displayed the latter [25]. Moreover, several studies showed differences in susceptibility to ischemia-reperfusion and albumin overload between male and female mouse kidneys [26–29]. The differences found between the mouse strains in our study in spontaneous proteinuria as well as proteinuria induced by presumed CPF-containing PP effluent further demonstrate the importance and influence of genetic background in the establishment of mouse models for CPF-induced proteinuria and FSGS.

Thy-1.1 transgenic mice spontaneously develop proteinuria and are susceptible to accelerated proteinuria due to administration of a monoclonal antibody against the Thy-1.1 protein. The Balb/c^Thy-1.1^ male demonstrated to be the most suitable genetic background, as substantially more mice responded to patient PP effluents compared to the other backgrounds we evaluated. However, as stated, there was no response to presumably CPF-containing serum or plasma. In order to examine potential differences in response to PP effluent and serum/plasma, simultaneously collected PP effluent, plasma, and serum were compared, but all these samples tested negative. Unfortunately, there was no serum or plasma collected along with the PP effluents that did induce a response. Evidently, there are differences between PP effluent, plasma, and serum that could influence either the concentration of CPF(s) and/or the ratio of CPF(s) to other constituents (e.g. CPF inhibitor) that could affect its activity [9]. Plasma obtained via PP differs from plasma collected via whole blood. Furthermore, it has been shown that contact of the patient body to the PP device alters the production of certain blood compounds, such as cytokines, which affects the composition of PP effluent compared to pre-PP serum/plasma [38]. Also, serum and plasma obtained from whole blood differ, because plasma is obtained via centrifugation after adding anticoagulants, while serum requires clotting first, thereby extracting many coagulant proteins and proteins nonspecifically bound to clotting proteins, or randomly captured during clot formation. Moreover, proteomic and lipidomic studies have demonstrated multiple differences between serum and plasma [39–41].

In summary, this study aimed to develop an *in vivo* model with proteinuria as a robust clinically relevant and easily measurable and quantifiable readout, for detecting the presence of CPF(s) in biological material from patients with pFSGS, ideally applicable to serum, in order to predict recurrence after transplantation. We observed proteinuria responses to presumably CPF-containing PP effluents, but not to sera. We have to conclude that the Balb/c^Thy-1.1^ male mouse strain as currently established is not suitable for detecting CPF(s) in serum from patients with pFSGS for diagnostic purposes in a clinical setting. However, a mouse model is more clinically relevant than *in vitro* models and thus our Balb/c^Thy-1.1^ males could have value as research models, e.g. to study CPF(s) in PP effluent that did induce a proteinuric response, or to review more subtle e.g. histological changes induced by CPF(s).

## Supporting information

S1 Fig Development of spontaneous proteinuria of Thy-1.1 transgenic mouse strains. Urine was collected weekly and urinary albumin was analyzed of (**A**) Balb/c^Thy-1.1^ males, (**B**) Balb/c^Thy-1.1^ females, (**C**) C57BL/6^Thy-1.1^ males, (**D**) C57BL/6^Thy-1.1^ females, (**E**), 129X1/Sv^Thy-1.1^ males, (**F**) 129X1/Sv^Thy-1.1^ females, (**G**) 129S2/SvPas^Thy-1.1^ males, (**H**) 129S2/SvPas^Thy-1.1^ females, (**I**) FVB/N^Thy-1.1^ males, and (**J**) FVB/N^Thy-1.1^ females.
(DOCX)
