# Supplementary material for: Novel mouse strains to study circulating permeability factor(s) in primary focal segmental glomerulosclerosis
Source: PLoS One. 2022 Sep 22;17(9):e0274959. doi: 10.1371/journal.pone.0274959 (PMC9499224; doi:10.1371/journal.pone.0274959)
Supplement: S1 Fig — Urine was collected weekly and urinary albumin was analyzed of (A) Balb/cThy-1.1 males, (B) Balb/cThy-1.1 females, (C) C57BL/6Thy-1.1 males, (D) C57BL/6Thy-1.1 females, (E), 129X1/SvThy-1.1 males, (F) 129X1/SvThy-1.1 females, (G) 129S2/SvPasThy-1.1 males, (H) 129S2/SvPasThy-1.1 females, (I) FVB/NThy-1.1 males, and (J) FVB/NThy-1.1 females. (DOCX) [file pone.0274959.s001.docx]

**

**

**S1 Fig. Development of spontaneous proteinuria of Thy-1.1 transgenic mouse strains.** Urine was collected weekly and urinary albumin was analyzed of (**A**) Balb/c^Thy-1.1^ males, (**B**) Balb/c^Thy-1.1^ females, (**C**) C57BL/6^Thy-1.1^ males, (**D**) C57BL/6^Thy-1.1^ females, (**E**), 129X1/Sv^Thy-1.1^ males, (**F**) 129X1/Sv^Thy-1.1^ females, (**G**) 129S2/SvPas^Thy-1.1^ males, (**H**) 129S2/SvPas^Thy-1.1^ females, (**I**) FVB/N^Thy-1.1^ males, and (**J**) FVB/N^Thy-1.1^ females.
